# Research on Monitoring Methods for Electrostatic Discharge Pulses in Spacecraft Dielectric Materials

**DOI:** 10.3390/mi16020180

**Published:** 2025-01-31

**Authors:** Hong Yin, Cunhui Li, Chengxuan Zhao, Xiaogang Qin, Xiaojin Lu, Xuan Wen, Liang Shi, Qing Liu, Jun Wang, Hanwu Jia, Shengsheng Yang

**Affiliations:** 1 Science and Technology on Vacuum Technology and Physics Laboratory, Lanzhou Institute of Physics, Lanzhou 730000, China; 2 School of Integrated Circuits, Xidian University, Xi’an 710000, China; 3 The 43rd Research Institute of China Electronics Technology Group Corporation, Hefei 230000, China

**Keywords:** spacecraft electrostatic discharge, FR4 (Flame-Retardant 4) dielectric materials, monopole antenna, impedance matching, standing wave ratio, real-time monitoring

## Abstract

Space particle radiation induces charging and discharging phenomena in spacecraft dielectric materials, leading to electrostatic discharge (ESD) and electromagnetic pulses (EMP), which pose significant risks to spacecraft electronic systems by causing interference and potential damage. Accurate and timely monitoring of these phenomena, combined with a comprehensive understanding of their underlying mechanisms, is critical for developing effective protection strategies against satellite charging effects. Addressing in-orbit monitoring requirements, this study proposes the design of a compact sleeve monopole antenna. Through simulations, the relationships between the antenna’s design parameters and its voltage standing wave ratio (VSWR) are analyzed alongside its critical performance characteristics, including frequency band, gain, radiation pattern, and matching circuit. The proposed antenna demonstrates operation within a frequency range of (28.73–31.25) MHz (VSWR < 2), with a center frequency of 30 MHz and a relative bandwidth of 8.4%. Performance evaluations and simulation-based experiments reveal that the antenna can measure pulse signals with electric field strengths ranging from (−1000 to −80) V/m and (80 to 1000) V/m, centered at 25.47 MHz. It reliably monitors discharge pulses generated by electron irradiation on spacecraft-grade FR4 (Flame-Retardant 4) dielectric materials, providing technical support for the engineering application of discharge research in space environments.

## 1. Introduction

During orbital deployment, spacecraft materials and components are inevitably exposed to various environmental stressors, including space plasmas, intense charged particle radiation, and solar radiation. These factors collectively contribute to radiation damage, which manifests in diverse forms such as total dose effects, single-event effects, and, most critically, charging and discharging phenomena [1,2]. Such effects significantly influence the reliability, lifespan, and overall success of spacecraft missions. Among these, particle radiation-induced charging and discharging within spacecraft dielectric materials pose a severe threat to spacecraft integrity and operational safety [1,2,3]. The phenomenon of Spacecraft Charging-Induced Electrostatic Discharge (SESD) encompasses two key processes. The first involves the interaction of space plasmas, high-energy electrons, and other environmental factors with dielectric materials, resulting in the gradual accumulation of electrostatic charges and the establishment of the electric field within and around the material. The second occurs when the intensity of this electric field exceeds the dielectric material’s breakdown threshold, initiating a charge-discharge process. On the external surfaces of dielectric materials, electrostatic discharge can take the form of corona discharge, flashover, or breakdown. Within the bulk of the dielectric material, discharge occurs when the rate of charge accumulation surpasses the rate of dissipation, creating an internal electric field that exceeds the material’s breakdown strength. This rapid discharge event releases substantial charges and electromagnetic pulses (EMP) within a short duration, posing risks of damage to satellite electronic systems and potentially causing sustained discharge in high-voltage components through multiple pathways [4,5]. Consequently, a detailed investigation of satellite charging and discharging mechanisms, alongside the development of real-time, accurate in-orbit monitoring systems, is essential. These efforts serve as fundamental prerequisites for the comprehensive evaluation and design of effective protection strategies against spacecraft charging and discharging effects. Such advancements are critical for ensuring the reliability, safety, and mission success of spacecraft operating in the harsh space environment.

SESD can generate high voltage, strong electric fields, and transient heavy currents, accompanied by intense electromagnetic radiation, resulting in an electrostatic discharge (ESD) electromagnetic pulse. This pulse can directly penetrate electronic devices or couple into sensitive circuits within equipment through pathways such as holes, slots, and cables, leading to damage or malfunction of these circuits. Such effects not only cause severe interference and damage to electronic equipment but also compromise the operational reliability of the satellite platform, posing significant risks to the safe and stable operation of satellites in orbit [6].

The current array of detection equipment for monitoring electrostatic discharge (ESD) electromagnetic radiation pulses in spacecraft includes monopole, dipole [7,8,9], TEM horn [10], log-periodic [11], long-wire, planar, flexible antennas, and planar interdigital sensors [12]. However, these systems face significant limitations in monitoring space discharge pulses, such as frequency band mismatches and excessive antenna sizes. The operating frequency bands of these antennas often fail to align with the primary energy frequency range of spacecraft ESD radiation, resulting in electromagnetic signals that do not accurately or comprehensively capture the radiation characteristics of ESD. Additionally, performance requirements, such as antenna gain and operating frequency, necessitate large antenna designs that are unsuitable for spaceborne applications [6,12,13,14]. For example, the monopole antenna described in [14] has a length of approximately 2 m, rendering it impractical for satellite-based ESD measurement missions. Consequently, optimizing antenna systems to achieve compact size and lightweight designs is critical to meet the stringent payload requirements of satellite missions.

Monopole antennas are characterized by their simple structure, omnidirectional reception, high-frequency adaptability, fast response, high reliability, and low cost, making them highly suitable for applications in space communications, telemetry and remote control, navigation and positioning, as well as electromagnetic environment monitoring and electrostatic discharge detection. For example, Ref. [15] proposed a multi-band U-shaped monopole antenna with resonant frequencies of 2.8 GHz, 5.8 GHz, and 10.8 GHz and impedance bandwidths of 880 MHz, 2500 MHz, and 3000 MHz, respectively; Ref. [16] designed a planar C-shaped monopole antenna with impedance bandwidths of 201 MHz, 330 MHz, and 1195 MHz in the 2.6 GHz, 3.5 GHz, and 5.5 GHz frequency bands, respectively, for global microwave access interoperability (WiMAX) applications; Ref. [17] designed a three-band E-type monopole antenna for WLAN communication applications at 2.4 GHz, 5.4 GHz, and 5.8 GHz; Refs. [8,18] proposed a monopole antenna deployment mechanism for VHF (144–146 MHz) and UHF (435–440 MHz) communication applications on the BIRDS-2 CubeSat. Monopole antennas are particularly suitable for spacecraft discharge pulse monitoring applications, where discharge time and orientation are difficult to predict, and pulse rise times are short. For instance, Ref. [19] designed a monopole antenna for electrostatic discharge from spacecraft solar cells. However, there are currently very few reports in the literature on in-orbit discharge monitoring experiments of dielectric materials prone to electrostatic charge accumulation using monopole antennas. The existing literature primarily describes antenna designs from simulation or simple experimental perspectives [14,15,16,17,18,19,20]. This lack of research has impeded the development of mature technologies and the systematic accumulation of data for spacecraft ESD monitoring systems.

Therefore, this paper, focusing on in-orbit flight applications, discusses the adaptive design and feasibility simulation test verification of monopole antennas for monitoring electrostatic discharge in spacecraft dielectric materials, providing technical guidance for the practical space application of such antennas. To meet the requirements of in-orbit flight experiments, the key technical specifications set for the pulse monitoring antenna in this study are as follows: the pulse electric field measurement range is (−1000~−80) V/m and (80~1000) V/m, with a lower limit of 80 V/m for electric field measurement; the center frequency for transient pulse measurement is 30 MHz ± 5 MHz. Based on these design objectives, research on small monopole antennas was conducted, investigating the effects of different antenna design parameters and matching circuits on antenna performance through simulation. This work aims to optimize the antenna design, conduct discharge pulse performance testing, and carry out in-orbit simulations for monitoring electrostatic discharge pulses from spacecraft dielectric materials, providing a reference for the implementation of discharge pulse detection in spacecraft and strengthening spacecraft protection technologies.

## 2. Simulation-Based Structural Design and VSWR Analysis of Monopole Antennas for Spacecraft ESD Monitoring

### 2.1. Principles and Structures of Monopole Antennas

**Figure 1:**
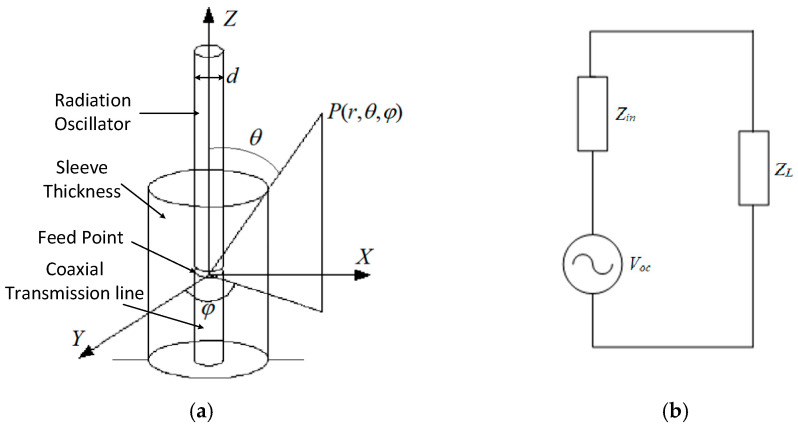
Structural and circuit characteristics of the monopole antenna: (**a**) schematic of the sleeve monopole antenna structure; (**b**) equivalent receiving circuit diagram of the antenna.

Based on the discharge pulse characteristics discussed earlier, we designed and optimized the monopole antenna. As illustrated in Figure 1a, the monopole antenna consists of three primary components: the radiating element, the sleeve, and the coaxial transmission line. The outer surface of the radiating element functions as the receiver and radiator of electromagnetic waves, while the inner surface of the sleeve serves as the outer conductor of the coaxial transmission line [8]. In the spherical coordinate system, angular variables θ and φ define the direction, point p represents the radiation point, and r is the distance from the radiation point to the origin. The feed point of the sleeve monopole antenna is located within the sleeve, with the detected signals transmitted through a 50 Ω coaxial radio frequency (RF) cable to the backend detection circuitry [7,8,9].

The sleeve monopole antenna functions as a transducer that converts spatial electromagnetic wave energy into high-frequency current energy. Its measurement bandwidth is closely linked to the acquired electrostatic discharge (ESD) information. To meet the requirements of spatial ESD monitoring, the antenna should have as wide a frequency band as possible. In this design, the impedance bandwidth—defined as the frequency range where the VSWR falls below a specified value—is used as an indicator of the antenna’s performance.

Using equivalent circuit analysis (Figure 1b), the antenna can be modelled as a voltage generator comprising an ideal voltage source *V*_oc_ and an internal impedance *Z*_in_, where *Z*_in_ represents the antenna impedance and is expressed as *Z*_in_ = *R_in_* + *jX_in_*. Here, *Z*_L_ is the load impedance. When the monopole antenna’s maximum receiving direction aligns with the incoming wave direction, the antenna’s polarization matches that of the incoming wave, and Z_in_ is conjugate-matched to *Z*_L_ (*Z*_L_ = *R_in_* − *jX_in_*). Under these conditions, the receiving antenna operates optimally, maximizing the power delivered to the load $\left(P_{Lmax}^{2}=V_{oc}/8R_{in}\right)$.

The dimensions of the monopole antenna significantly influence its input impedance Z_in_, which in turn affects the antenna’s impedance bandwidth and load power P_L_. To achieve better radiation power from the antenna and improve the discharge pulse measurement results, apart from reducing transmission losses, it is essential to enhance the electromagnetic signal induction capability, which depends on the antenna’s structural dimensions. Therefore, for the detection of discharge pulses from spacecraft dielectric materials, it is necessary to investigate the factors influencing the performance of monopole antennas, including structural dimensions and matching circuits, to design a discharge pulse detection device suitable for spacecraft charging and discharging.

When an electrostatic discharge (ESD) pulse with an electric field intensity *E_i_* is incident on a monopole antenna of length *l* and radius *d*/2 at an angle *θ*, the equivalent source voltage *V_oc_*(*t*) corresponds to the open-circuit voltage *V_l_*(*t*) of the antenna. Here, *l_e_* represents the effective length of the antenna, defined as half the length of the radiating element.

(1) $$V_{oc(t)}=V_{l(t)}=E_{i}(t)\cdot l_{e}\cdot cos(\theta )$$

To account for the precision of the electronic system, the influence of system noise on measurements, and the effects of discharges on spacecraft during in-orbit operations, the antenna length *l* is set to 50 mm. Under these conditions, the minimum electric field intensity of space ESD detectable by the system is approximately 40 V/m.

### 2.2. Configuration and Modeling of Antenna Parameters

**Figure 2:**
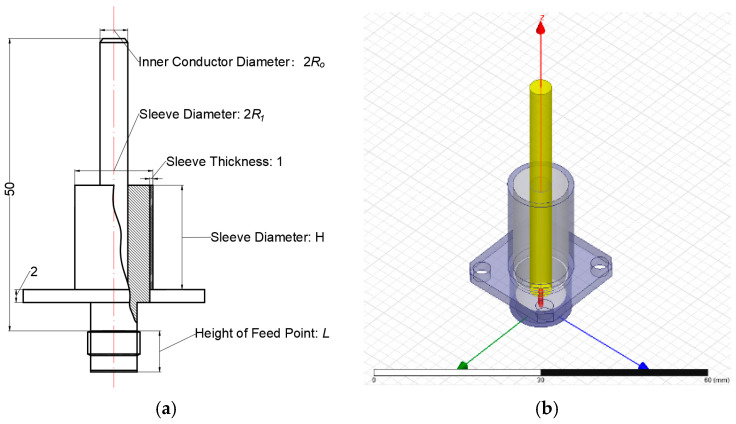
Basic dimensions and modeling of a sleeve monopole antenna: (**a**) cross-sectional diagram of the sleeve monopole antenna; (**b**) 3D model of the sleeve monopole antenna.

As shown in Figure 2a, the sleeve monopole antenna parameters include the height of the inner conductor within the sleeve (*H*_1_), the overall height of the sleeve (*H*), the height of the feed point (*L*), the radius of the inner conductor (*R*_0_), and the outer radius of the sleeve (*R*_1_). Specifically, *H*_1_ is set to 50 mm, and *R*_1_ is constrained to a maximum of 7.5 mm. The shaded area in the figure represents the polytetrafluoroethylene (PTFE) insulation layer, which has a minimum thickness of 2 mm. This layer provides structural support for the inner conductor and prevents short circuits.

To enable comprehensive analysis, four discrete values are assigned to each of the remaining four parameters as follows: *H* = {5 mm, 10 mm, 15 mm, 20 mm}; *L* = {3 mm, 4 mm, 5 mm, 6 mm}; *R*_0_ = {0.5 mm, 1 mm, 1.5 mm, 2 mm}; *R*_1_ = {3 mm, 4.5 mm, 6 mm, 7.5 mm}.

The antenna simulation and analysis of the sleeve monopole were performed using the parameters mentioned above. The antenna model is shown in Figure 2a, with its 3D representation shown in Figure 2b.

### 2.3. Simulation Results of VSWR

**Figure 3:**
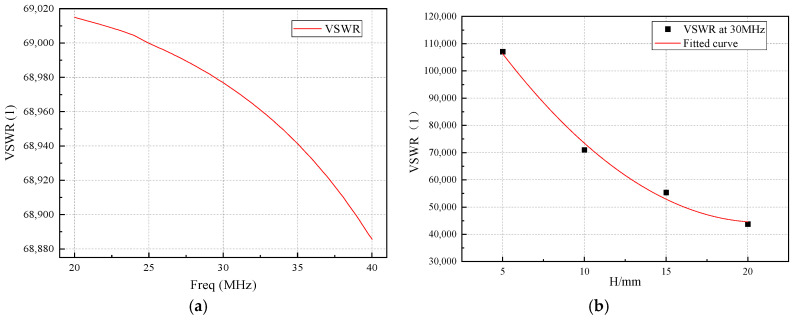
Simulation analysis of impact of frequency and sleeve height (*H*) variations on antenna VSWR: (**a**) impact of frequency variation (*L* = 4 mm, *R*_0_ = 2 mm, *R* = 6 mm, and *H* = 5 mm); (**b**) impact of sleeve height (*H*) variation (*L* = 3 mm, *R*_0_ = 1 mm and *R*_1_ = 4.5 mm).

Theoretical analysis of the sleeve monopole antenna predicts the first resonance point to occur around 1.5 GHz. However, the target center frequency for the antenna is 30 MHz, which is substantially lower than the first resonance point, leading to a voltage standing wave ratio (VSWR) significantly exceeding the typically required value of 5 for antennas. Using the parameter values *L* = 4 mm, *R*_0_ = 2 mm, *R*_1_ = 6 mm, and *H* = 5 mm as an example, the VSWR curve is illustrated in Figure 3a.

To analyze the relationship between the sleeve height (*H*) and VSWR, simulations were conducted by varying *H* while keeping other parameters constant. As shown in Figure 3b, increasing the sleeve height improves the VSWR characteristics, demonstrating a strong dependency of VSWR performance on *H*.

**Figure 4:**
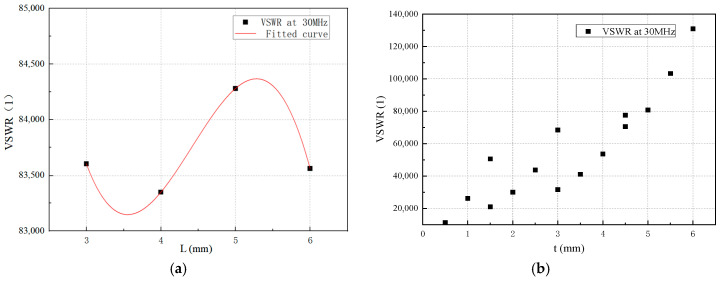
Simulation analysis of impact of feed point (*L*) and insulation layer thickness (*t*) variations on antenna VSWR: (**a**) impact of feed point height (*L*) variation (*H* = 15 mm, *R*_0_ = 0.5 mm, *R*_1_ = 4.5 mm); (**b**) impact of insulation layer thickness (*t*) variations (*H* = 20 mm, *L* = 3 mm).

As illustrated in Figure 4a, when the feed point height (*L*) is varied independently, optimizing the VSWR characteristics depends on the specific values of the other three parameters, with no consistent pattern observed. Additionally, the influence of *L* on the antenna’s VSWR is significantly less pronounced compared to the impact of *H*.

The antenna’s VSWR is also influenced by the thickness of the insulation layer (*t*) within the sleeve, defined as *t* = *R*_1_ − *R*_0_. Figure 4b demonstrates that a thinner insulation layer between the sleeve and the inner conductor generally results in improved VSWR performance. While isolated cases show better VSWR with a thicker insulation layer, the overall trend confirms that the radius of the inner conductor (*R*_0_) has a significant effect on the VSWR.

For space applications, where insulation performance is critical, the insulation layer thickness (*t*) is set to 2 mm. To investigate the relationship between *R*_0_ and VSWR performance, the following parameters are configured: *H* = 20 mm; *L* = {3 mm, 4 mm, 5 mm, 6 mm}; *R*_0_ = {0.5 mm, 1 mm, 1.5 mm, 2 mm, 2.5 mm} (limited by the maximum screw thread inner wall radius), *R*_1_ = *R*_0_ + 2 mm + 1 mm (where 2 mm is the insulation layer thickness and 1 mm is the sleeve thickness).

**Figure 5:**
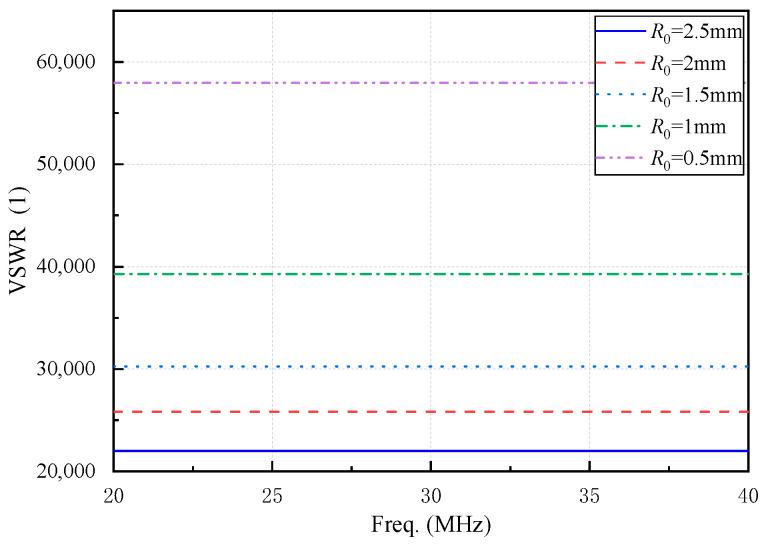
The curves of antenna VSWR in the case of *H* = 20 mm and *R*_1_ = *R*_0_ + 3 mm.

As shown in Figure 5, 20 VSWR curves are generated by combining four possible values of *L* and five possible values of *R*_0_. These curves exhibit a distinct pattern: variations in *R*_0_ create groups of four curves, each reflecting differences in *L*. The results indicate that increasing *R*_0_ enhances the VSWR performance of the sleeve monopole antenna, highlighting the significant influence of the inner conductor radius on overall performance.

**Figure 6:**
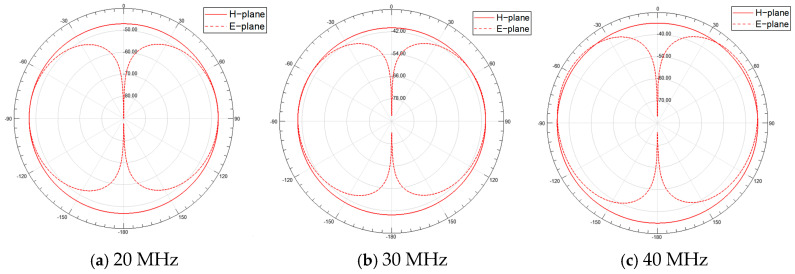
Radiation patterns of the sleeve monopole antenna: the radiation patterns at 20 MHz, 30 MHz, and 40 MHz are shown, with the solid line representing the H-plane and the dashed line representing the E-plane.

**Figure 7:**
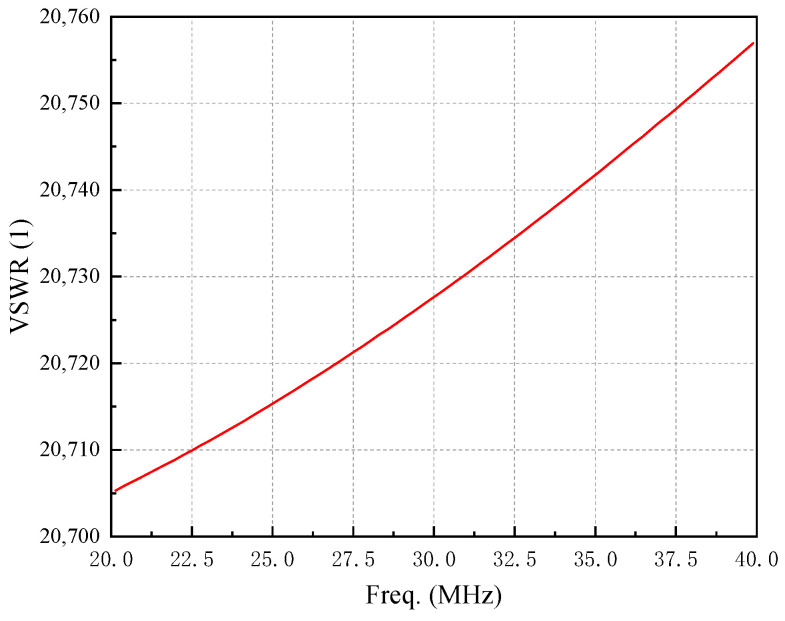
VSWR of the sleeve monopole antenna under optimal conditions.

The optimal VSWR performance of the sleeve monopole antenna is achieved with the following parameter configuration: *H* = 20 mm, *L* = 3 mm, *R*_0_ = 2.5 mm. Under these conditions, the radiation pattern and gain of the antenna are presented in Figure 6, where the solid line represents the H-plane and the dashed line represents the E-plane. The antenna exhibits omnidirectional radiation characteristics in the H-plane within the (20–40) MHz range, while the E-plane demonstrates strong radiation capability. The maximum gains for the three cases are −46.9 dB, −39.4 dB, and −33.5 dB, respectively. The corresponding VSWR results are shown in Figure 7.

As illustrated in Figure 7, the antenna with the optimal parameters demonstrates a significant impedance mismatch, leading to considerable energy loss. To address this issue, an impedance-matching circuit must be designed based on the antenna’s port impedance at 30 MHz.

## 3. Design of Impedance Matching Circuit

**Figure 8:**
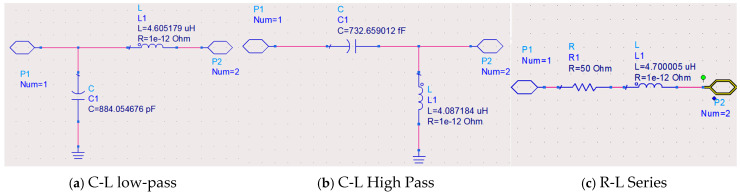
Antenna matching circuit diagram.

Under optimal VSWR conditions, the port impedance of the sleeve monopole antenna at 30 MHz is (0.71-862.14j) Ω and an impedance matching circuit for the antenna is designed. To minimize energy consumption within the matching circuit, only capacitors and inductors are used, although resistors may be introduced in specific cases to broaden the antenna’s VSWR bandwidth. As shown in Figure 8, based on matching circuit principles, three types of circuits were designed: C-L Low-pass L-Shaped Matching Circuit, C-L High-pass L-Shaped Matching Circuit, and R-L Series Matching Circuit.

**Figure 9:**
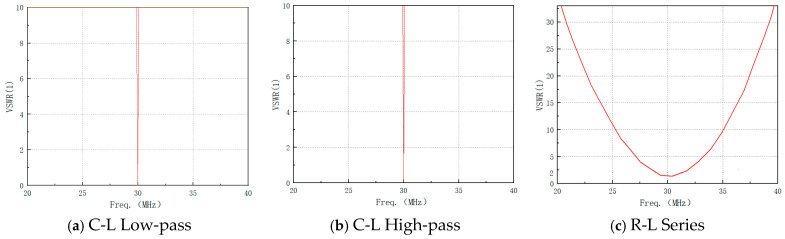
Simulated values of antenna VSWR under different matching circuits.

As shown in Figure 9, when the C-L Low-pass L-shaped matching circuit is introduced, the sleeve monopole antenna achieves a VSWR below two within the frequency band (29.96–30.03) MHz. At 30 MHz, the circuit achieves perfect matching without introducing losses. Additionally, losses are reduced compared to the antenna without the matching circuit. Similarly, with the C-L High-pass L-shaped matching circuit, the VSWR remains below 2 in the frequency band (29.98–30.02) MHz. Perfect matching at 30 MHz again results in no circuit losses, with reduced losses. However, the practical implementation of L-shaped matching circuits is hindered by the challenges of fabricating femtofarad-level capacitors, making these circuits difficult to realize in practice.

Although L-shaped matching circuits effectively reduce the VSWR below two around 30 MHz, their bandwidth remains limited. For example, the C-L low-pass L-shaped matching circuit achieves a bandwidth of only 0.088 MHz, corresponding to a relative bandwidth of 0.23% (see Table 1). To address this limitation and achieve broader bandwidth, resistors must be incorporated into the matching circuit.

Considering the practical limitations in the manufacturing and procurement of components such as resistors and inductors, as well as the demand for bandwidth, the R-L series matching circuit is configured with a resistance *R* = 50 Ω and an inductance *L* = 4.7 μH for simulation. This design extends the operating frequency band of the sleeve monopole antenna to (28.73~31.25) MHz (the VSWR remains below 2), resulting in a relative bandwidth of 8.4%. Consequently, the R-L series matching circuit is selected as the optimal design due to its superior bandwidth performance and practical feasibility.

## 4. Experimental Verification of Discharge Pulse Detection

To evaluate the discharge pulse detection capability and spatial adaptability of the designed antenna and matching circuit, performance tests were conducted. These included discharge pulse detection experiments and simulation tests to measure the discharge characteristics of spacecraft dielectric materials.

### 4.1. Discharge Pulse Performance Testing of the Antenna

**Figure 10:**
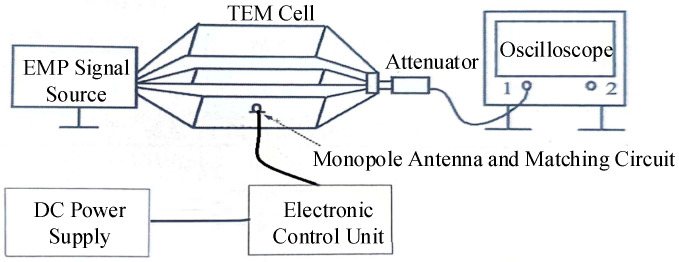
System layout for discharge pulse performance testing of the antenna.

The discharge pulse performance testing system is illustrated in Figure 10 and consists of the following components: an EMP signal source, a TEM (Transverse Electromagnetic Wave) cell, an attenuator, an oscilloscope, a DC power supply, a control unit, a monopole antenna, and ground testing equipment. The EMP signal source consists of a high-precision pulse generator and a power amplifier and is used in conjunction with the self-developed TEM cell, a parallel plate test chamber designed to simulate a uniform electric field. The attenuator regulates signal strength to prevent downstream equipment damage. The control unit collects data from the antenna and manages the system state.

The experiment is divided into two parts. The first part involves the calibration of the electric field inside the TEM cell. A standard square pulse signal with a rise time of 20 ns is generated by the EMP signal source and the voltage signal $U_{1}$ inside the TEM cell is recorded using an oscilloscope. The transient electric field amplitude *E* in the EMP cell is then calculated using the following formula:

(2) $$E=U_{1}\cdot \alpha /h$$

where α is the attenuation factor of the attenuator, and *h* is the spacing between the TEM cell plates. During the test, the output voltage amplitude of the EMP signal source is adjusted point by point to achieve the desired field strength. The actual field strength is calculated using the measured voltage $U_{1}$, allowing for the calibration of the electric field inside the TEM cell within the range of −1000 to 1000 V/m.

The second part involves measuring the electric field measurement performance of the pre-fabricated antenna using the calibrated standard electric field in the TEM cell. The ADC code range of the Electronic Control Unit is 0~4095, and its trigger threshold is set to below 50 V/m. Within the range of −1000 to 1000 V/m, electric field values are incrementally selected, and the electric field in the EMP cell is adjusted for each test point. Pulse measurement experiments are conducted at each electric field point, and the transient electric field amplitude *E* is correlated with the ADC code values corresponding to the peaks of the output pulse waveforms.

**Figure 11:**
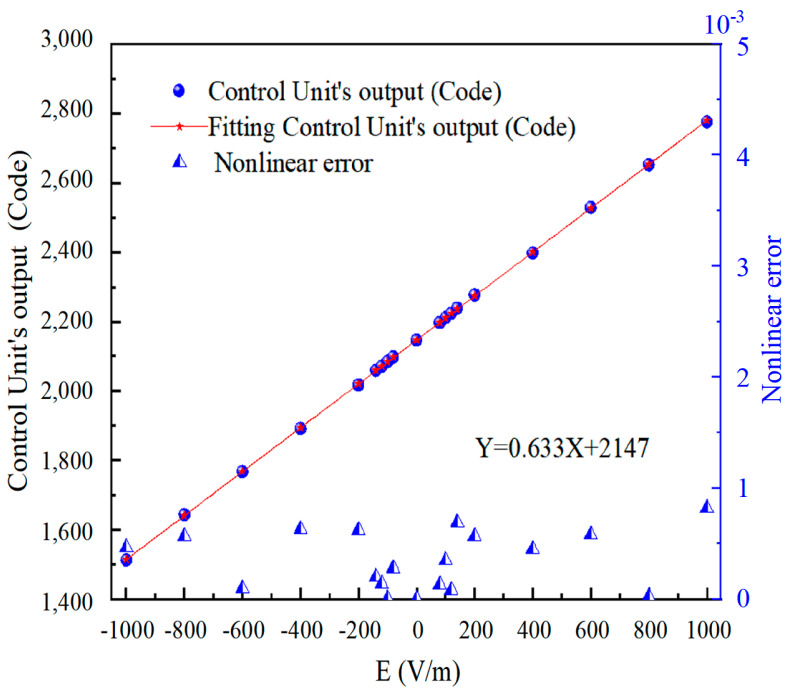
Response test results of the monopole antenna to the standard electric field.

The experimental results are summarized in Figure 11, where linear regression fitting and linear error analysis were performed on the input and output data. (The nonlinear error is defined as the maximum deviation between the output–input curve and its fitted linear curve, typically expressed as $\Upsilon_{V}=\left(∆V/V_{Fs}\right)\times 100\%$, where ∆V represents the maximum nonlinear deviation of the output, and $V_{Fs}$ is the full-scale output [21]). It can be observed that the output of the pre-fabricated antenna and control unit exhibits a good linear relationship with the input electric field, with the maximum linear error being less than 0.1%, enabling electric field measurements within the ranges of (−1000~−80) V/m and (80~1000) V/m. Additionally, the frequency sweep range of the network analyzer was set to 0–50 MHz, and the measured center frequency of the antenna was found to be 25.47 MHz, meeting the design requirements.

### 4.2. Simulation Experiments for Discharge Detection of Spacecraft Dielectric Materials

**Figure 12:**
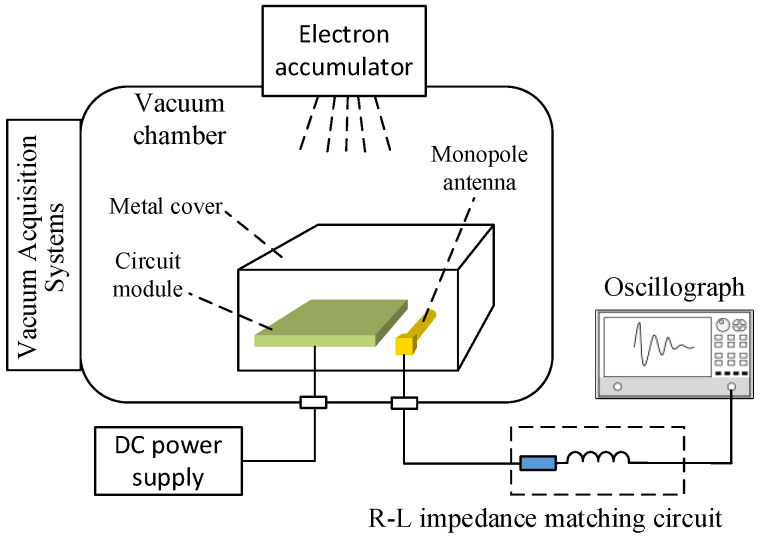
Schematic diagram of satellite discharge pulse detection simulation experiment.

As shown in Figure 12, the aluminum metal cover, which houses the monopole antenna and aerospace-grade FR4 substrate circuit module, is placed inside the electronic accelerator irradiation chamber. The aluminum box, with a certain thickness, simulates the spacecraft’s skin and outer shell. The electrical ground of the circuit module is connected to the metal cover, while the metal cover is isolated from the earth ground through a high-resistance insulator. The DC power supply and oscilloscope are connected to the main power through a series isolation transformer to complete the power supply. The above design simulates the isolated suspension of the spacecraft and the electrical ground connection state of the spacecraft’s internal circuits.

In this experiment, electron beams with different beam current densities generated by a 2 MeV electron accelerator were used to irradiate the test object under a vacuum pressure of 1 × 10^−5^ Pa, simulating the charge and discharge behavior of FR4 dielectric material in the circuit board of a spacecraft under typical space radiation conditions. The self-designed monopole antenna was installed adjacent to the circuit module within the insulated aluminum housing to detect discharge pulses resulting from electron irradiation. The antenna was connected to an R-L impedance matching circuit and interfaced with a high-speed oscilloscope for real-time waveform capture and output.

**Figure 13:**
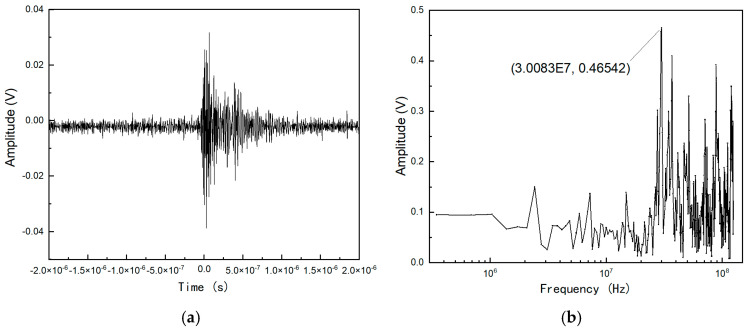
Discharge pulse characteristics of FR4 dielectric material for satellites: (**a**) temporal characteristics of signals; (**b**) spectral characteristics of signals.

Figure 13 presents partial results of the discharge pulse detection test and their corresponding frequency spectrum diagrams. The results confirm that the designed antenna effectively and stably receives electromagnetic signals emitted from FR4 material discharges, with strong signal acquisition near 30 MHz. Statistical analysis revealed that the rise times of the captured pulse signals ranged from 10 to 40 ns, while the pulse widths were generally between 5 and 12 μs. Discharge pulses were detected after a period of electron irradiation, regardless of whether the spacecraft circuit module was powered or unpowered. The measured discharge occurrences are summarized in Table 2. At an electron beam current density of 0.16 pA/cm^2^, the interval between discharge pulses was approximately 30 min. As the current density increased to 32 pA/cm^2^, the interval decreased to less than 10 min. This behavior demonstrates that the charge accumulation and dissipation intervals of FR4 material are inversely proportional to the beam flux: higher beam flux results in shorter intervals between discharge pulses. This is attributed to the increased deposition of charges on the FR4 material, making discharge events more frequent. The experimental results validate the stable signal acquisition capabilities of the designed antenna, demonstrating its suitability for monitoring and evaluating electromagnetic emissions associated with dielectric material discharges in spacecraft applications.

## 5. Conclusions

Based on the results and discussions presented, the following conclusions are drawn:

(1) A sleeve monopole antenna tailored for monitoring the electrostatic discharge (ESD) processes in spacecraft dielectric materials was successfully designed. The antenna operates in the frequency range (28.73~31.25) MHz (VSWR < 2), with a center frequency of 30 MHz and a relative bandwidth of 8.4%.
(2) The antenna’s VSWR performance improves with an increase in sleeve height (*H*), an increase in the inner conductor radius (*R*_0_), and a decrease in the insulation layer thickness (*t*) between the sleeve and the inner conductor. The feed point height (*L*) also impacts VSWR performance but is dependent on its interaction with other parameters. Among the three impedance matching circuits designed, the R-L series matching circuit demonstrated superior bandwidth characteristics, outperforming the other configurations.
(3) Experimental verification showed that the antenna is capable of measuring pulse signals with electric field strengths in the ranges (−1000 to −80) V/m and (80 to 1000) V/m. With a measured center frequency of 25.47 MHz, the antenna reliably monitored discharge pulses generated by electron irradiation on spacecraft-grade FR4 dielectric materials, fulfilling the design requirements.

This study comprehensively investigates the application of monopole antennas in space electrostatic discharge (ESD) monitoring through methods such as antenna simulation design, performance testing of antenna prototypes, and simulated experiments for spacecraft dielectric material discharge measurements. The findings offer valuable engineering guidance for the practical space application of such antennas and will address the current lack of empirical support for the use of monopole antennas in space ESD testing.

## Figures and Tables

**Table 1:** Overview of antenna matching circuit performance.

Matching Network Type	C (pF)	L (µH)	R (Ω)	Bandwidth Characterization (MHz) (VSWR < 2)	Relative Bandwidth
C-L Low-pass	884	4.6	/	29.96–30.03	0.23%
C-L High-Pass	0.733	4.1	/	29.98–30.02	0.13%
R-L Series	/	4.7	50	28.73–31.25	8.4%

**Table 2:** FR4 dielectric materials discharge behavior under varied electron beam irradiation.

Beam Density (pA/cm^2^)	Time (min)	Number of Discharges (Times)
0.16	0~30	1
	30~90	2
	90~180	4
0.81	0~30	1
	30~90	3
	90~180	5
4.2	0~30	2
	30~90	4
	90~180	7
19	0~30	2
	30~90	6
	90~180	9
32	0~30	3
	30~90	7
	90~180	11

## Data Availability

Data are contained within the article.
